# Beyond pollination syndromes? Reflections on the classifications of Federico Delpino

**DOI:** 10.1093/aob/mcaf197

**Published:** 2025-08-25

**Authors:** Quentin Cronk

**Affiliations:** Department of Botany and Beaty Biodiversity Museum, University of British Columbia, Vancouver, BC, Canada V6T 1Z4

**Keywords:** Pollination biology, pollination ecology, pollination syndrome, floral functional type, floral functional trait, Federico Delpino

## Abstract

**Background:**

Pollination syndromes are typically defined as idealized pollination modes based on a canonical set of traits. As such, they are often criticized as typological rather than empirical. These syndromes are attributed to the great Italian botanist Federico Delpino, who has borne some blame for their perceived shortcomings. Yet Delpino’s original contribution, although it inspired the concept of pollination syndromes, differed significantly from it. What he proposed was, in contrast, an empirical classification of plants into floral functional types (FFTs), with pollination vector as the key trait.

**Perspective:**

Delpino produced two functional type classifications. The first was published, approvingly, by H. Müller in 1873. This was the classification that, in the 20th century, evolved into the pollination syndrome concept. Delpino later proposed a more ambitious and innovative classification of animal pollination into 47 functional types in his monumental work, the *Ulteriori Osservazioni*. Müller strongly criticized this classification, but if Delpino’s second scheme had been refined rather than dismissed, it might have shaped later developments in pollination biology in a highly beneficial way. As Löw wrote in 1895, it was ‘one of the most ingenious and grand attempts’ at a fundamentally open-ended problem. Müller proposed his own classification in 1881, but after that, interest in floral functional types in pollination ecology languished, eventually to be replaced by a different concept, that of pollination syndromes. In contrast, plant functional type (PFT) classification has become central in vegetation and global change ecology.

**Conclusion:**

Pollination ecology could usefully reflect on the PFT approach, even revisiting Delpino’s work (which has never been translated from the original Italian) for inspiration. For some studies, an FFT approach, as pioneered by Delpino, could usefully replace a pollination syndrome-based approach.

## INTRODUCTION

This Botanical Briefing will argue that pollination biology should transition away from the use of the pollination syndrome concept to the alternative concept of floral functional types (FFTs), pioneered by the 19th century plant biologist Federico Delpino, who classified plants into 47 FFTs. His work, however, has been underappreciated, in part due to a failure to translate it from the original Italian and in part due to unwarranted attacks by his contemporary Herman Müller.

The concept of pollination syndromes has had a huge influence on the field of pollination biology. They are defined here as ‘an idealized characterization of one trait (pollination mode) on the basis of a canonical set of morphological traits’. They therefore tend to be conceptual and typological rather than empirical and have been criticized for that reason. The great 19th century Italian botanist Federico Delpino is often credited with inventing them. However, things are a bit more complicated. He did not invent the concept of pollination syndromes, although he provided the inspiration for them. He and his contemporaries regarded his work as providing a classification of plants into what we can now call FFTs, with pollination mode being the leading trait. It was the 20th century that transformed this into typological pollination syndromes, a set of morphologically defined pollination modes.

This may seem a minor transformation, but logically it is inverted. We can compare and contrast the two by the following pair of definitions. A *pollination syndrome* is a set of floral traits (presumed to be co-adapted for a hypothetical type of pollinator, such that the actual pollinator can be predicted from these traits). By contrast, a *floral functional type* is a set of species that share a set of floral traits related to pollination function (the actual pollinators being included in the set of traits).

It is perfectly reasonable to want to classify plants according to floral function. However, the way this is done via pollination syndromes is unusual to say the least. Here, the group (syndrome) is actually a conceptualization relating pollination mode to a set of canonical traits that are said to define it. A problem arises if a plant has a mismatch between the observed pollination mode and the morphological traits that are said to define the mode. However, this is no problem for a plain FFT classification: a new group can be created, or the variation can be incorporated into the description. ‘Pollination vector’ becomes just one trait of many, and can be described as variable (covering generalist pollination). Whereas classifications are robust to variation and can incorporate it, the pollination syndrome typology seems almost designed to be compromised by biological variation. Classifications, on the other hand, are empirical frameworks that perform an important practical function of organizing knowledge about variation.

That is not to say that the pollination syndrome typology is not a useful way of exploring pollination ecology. In fact, although the value of pollination syndromes has been extensively questioned (Waser *et al*., 1996; Ollerton *et al*., 2009), there has also been a defence (Fenster *et al*., 2004; Johnson and Wester, 2017; Dellinger, 2020). The fact that pollination syndromes are so frequently and continually discussed in the descriptive literature of pollination shows that they have considerable utility in framing the discussion of pollination mechanisms. An FFT classification might do even better.

How did we get here? To understand why pollination syndromes have been so hugely influential in pollination ecology, whereas FFTs have been ignored, we need a historical perspective. It turns out that the 19th century classifications put forward by Delpino and Müller were functional type classifications and not pollination syndromes as we know them today. It should have been a great start, but something extraordinary happened in the 20th century. The classification of plant functional types (PFTs) transformed into a classification of processes (pollination by particular agents), and the names were altered from (e.g.) anemophils (the wind pollination group) to anemophily (wind pollination, a syndrome), with the traits now applied to the processes, not the groups. The resulting shift – from classifying plant groups to typifying pollination processes – is profound, as it leads to something more conceptual, less empirical and hence more typological. We cannot attribute this to Delpino or Müller, as the change happened gradually over the 20th century, through the works of authors such as Werth, Vogel and van der Pijl (Werth, 1915; Vogel, 1954; van der Pijl, 1961). It was finalized decisively, though, at least in English, by Faegri and van der Pijl’s landmark 1966 book (Faegri and van der Pijl, 1966).

## THE 19TH CENTURY FFT CLASSIFICATIONS

In the late 19th century, three important FFT classifications were produced:

1. Delpino’s first classification, published in Müller’s 1873 book;
2. Delpino’s second classification, a much more granular account of animal pollination with 47 floral types; and
3. Müller’s own FFT classification (in his second major work, *Alpenblumen*), based on pollinator rewards and their placement (Müller, 1881).

**Fig. 1.**
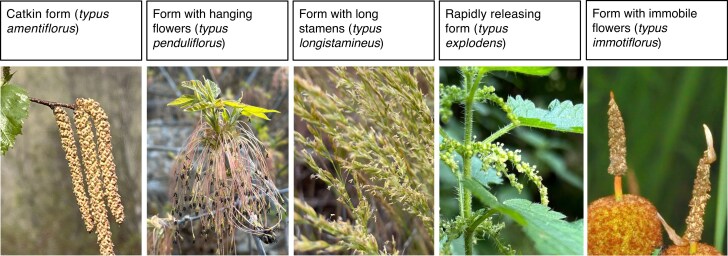
The five basic types (*typus*) of Division Anemophilae (plants with wind-pollinated flowers) in Delpino’s first functional classification (Supplementary Data Tables S1 and S2). Left to right: *Betula occidentalis*, *Acer negundo*, *Poa secunda*, *Urtica dioica* and *Typha minima*. All photos: Q.C., except *Typha minima* (credit: Hectonichus, CC BY-SA 3.0, Wikimedia Commons).

Delpino and his contemporaries called these ‘classifications’, and like any biological classification, they are empirically based. It was Delpino’s first, and high-level, classification (Supplementary Data Tables S1 and S2; Fig. 1) that was later, by others, turned into ‘pollination syndromes’. It was reproduced in full for the first time (and without criticism) by Müller in his first major work (Müller, 1873), and is known in English mainly from the translation of this work, at Darwin’s urging, by D’Arcy Thompson (Müller, 1883). Müller wrote: ‘Delpino has described the pollination mechanism of a great number of flower forms from various plant families … he has directly observed pollinators … and he ultimately selects the distinction of pollen transport agents as the primary criterion for classification … the following classification has been supplied to me by Delpino, by letter.’ The resulting classification of PFTs, Müller faithfully reproduces. Müller clearly approved of this classification and even added examples for it. The classification has three levels: first Hydrophilae, Anemophilae (Fig. 1) and Zoidophilae. Delpino/Müller then divide the zoidophils into Ornithophilae, Malacophilae and Entomophilae. Finally, the entomophils are divided into eight groups: Melittophilae (Melittophils), Micromelittophils, Myiophils, Micromyiophils, Sapromyiophils, Cantharophils, Psychophils and Sphingophils (Table S1).

The word ending is significant: -philae is a plural noun ending (i.e. a group, the ornithophils, the myophils, etc.). It is not the ending -phily, which indicates a process or concept, bird pollination, etc., as in pollination syndromes. It should therefore be stressed that these are plant functional groups, determined empirically by the presence or absence of various floral traits of functional significance, of which pollination mode is only one (but, in this case, the primary one). It is an intellectual predecessor to the PFTs (based on plant traits; Table 1) that have been developed over the last 40 years in macroecology, but the FFTs of Delpino pre-date PFTs by 100 years. We are now so familiar with functional type classifications that it is hard to appreciate just how innovative this was.

**Table 1.** Functional type definitions in macroecology following McMahon *et al*. (2011) and in pollination ecology (proposed).

	Macroecology	Pollination ecology
Syndrome	–	Pollination syndromes: idealized definitions of processes (pollination mode) based on putative defining traits
Functional type	Plant functional type (PFT): a group of species that display similar adaptations to environmental factors or perform similar functions within an ecosystem. PFTs are generally defined using plant functional traits related to, for example, life form, leaf form, phenology and bioclimatic limits.	Floral functional type (FFT): a group of species that show similar adaptations to pollination factors or other aspects of floral biology; defined using observed morphological and physiological traits as well as observed traits relating to pollinator–flower interaction.
Functional trait	Plant functional traits: measurable characteristics of plants that are believed to be diagnostic of physiological adaptations to environmental stressors, and are thus used to identify groups of species (or PFTs) that have specific advantages in given environments. Examples include leaf mass per area (or specific leaf area), wood density and seed mass.	Floral functional traits are measurable or scorable characteristics of flowers that are believed to be diagnostic of adaptive aspects of floral biology including pollination biology. These are used to identify groups of species (FFTs) that have shared floral adaptations, which may be correlated with pollination mode. Examples are: reward provision (pollen, nectar), perianth colour and morphology, dichogamy, and herkogamy.

Delpino’s second classification came out in the *Ulteriori osservazioni* (Delpino, 1873–1874). It was much more ambitious, with 47 floral types. Müller took exception to several of the groupings in two severe critiques (Müller, 1874, 1882). The *Ulteriori osservazioni* is not often read in the original Italian, but is known through Paul Knuth, who included the briefest of summaries in his widely read *Handbuch* (Knuth, 1898), and this notice came into English in the translation of Knuth’s handbook by J. R. Ainsworth Davis (Knuth, 1906). Despite the criticism, there are many very interesting ideas and observations contained in it, and we can say with Ernst Löw that for its time it was ‘one of the most ingenious and grand attempts to solve a problem that, by its nature, can never be fully resolved but will always require improvement and supplementation depending on the changing standpoint of knowledge’ (Loew, 1895). And that is the essence of a classification: it can be revised, extended and changed very easily (unlike pollination syndromes).

Delpino’s *Ulteriori osservazione* (including the 93-page classification) was an extraordinary intellectual achievement. Unfortunately, it appears to have been little read (although often cited). There are two main reasons for this. The first was Müller’s hostility to the classification, and the second was its relative obscurity; the two fascicles were hard to obtain, and it was never translated from the original Italian.

## MÜLLER’S CRITIQUE OF DELPINO’S SECOND FFT CLASSIFICATION

Just after Delpino had published the second classification in *Ulteriori osservazioni*, Müller summarized the latest pollination publications for a German journal (Müller, 1874), summarizing the whole of Delpino’s 47-type classification, and, in a series of footnotes, was highly critical of some of the groupings. He wrote: ‘D. believes that the seemingly inexhaustible diversity of floral structures can be organized into a specific number of types … it is, by the nature of the matter, entirely at the discretion of the author to consider a greater or lesser number of characteristics as decisive for grouping into a type, and a closer examination of the following 47 types, divided into 13 classes, clearly shows that with the same justification, 470 or 4700 different types could have been established.’ This raises the age-old question of whether it is possible to build a classification where there is no natural grouping agent like similarity by descent. Yet vegetation types, PFTs and biochemicals have all been classified, and those classifications are considered essential frameworks in the relevant branches of knowledge. By that standard, it is plainly wrong for Müller to imply that the classification of FFTs is impossible and useless. As to whether it should be 47 or 470 types, this is determined by the utility for the task at hand. Some applications will benefit from a fine-grained classification (e.g. ontology classifications of gene function), while for others, a coarse classification is all that is required.

Müller’s more specific criticisms (which he placed in the footnotes) do not criticize the activity of producing a classification, but the fact that it is based on erroneous empirical facts (although in many of these cases Delpino was arguably in the right). Even 8 years later, he was not prepared to let it drop and returned to his critique in another article (Müller, 1882), again criticizing the classification and group composition. Waser *et al*. (2011) have translated and discussed this critique thoroughly, suggesting it presents a case against pollination syndromes. As the concept of pollination syndromes did not exist in their modern form at this time, and his criticism was of a classification unrelated to the construction of pollination syndromes, our response should be carefully nuanced.

These attacks did not go unnoticed by contemporaries. Paul Knuth quotes the passage as an example of Müller’s ‘severe judgement’ and Ernst Löw, the intellectual successor to Müller after the latter’s untimely death in 1883, came to Delpino’s defence, writing: ‘At the time of its publication, the *Ulteriori osservazioni* constitutes a sort of conclusive work in which the floral forms of the tropical world also, for the first time, found a coherent biological interpretation’ (Loew, 1895). Müller was well known to his contemporaries for his lack of patience with those with whom he disagreed. In the words of his obituarist in *Science*: ‘He had, however, little patience with inaccuracy in observation, and, both publicly and in private, criticised errors with vigor’ (Anon, 1883).

## DELPINO WAS RIGHT: THE *BORAGO*-TYPE

**Fig. 2.**
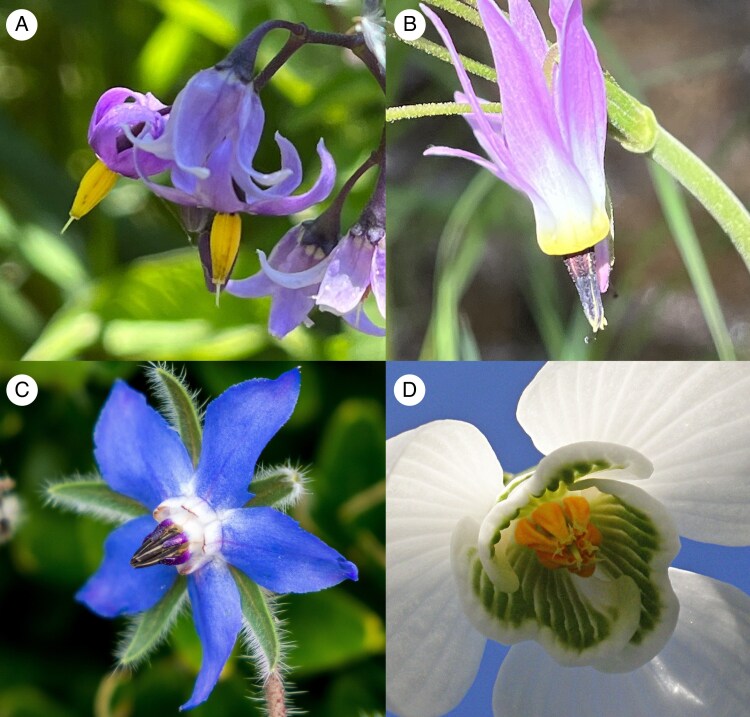
The ‘*Borago*-type’ functional group, as classified by Delpino. All members have an anther cone. In *Galanthus* the anther cone is loose, formed from connivent anthers. Delpino observed that the convergently evolved anther cones were all associated with being grasped by bees and with poricidal anther dehiscence, and hence they were grouped together functionally. (A) *Solanum dulcamara*; (B) *Primula pauciflora*; (C) *Borago officinalis*; (D) *Galanthus* sp. In A and B, the pollen is released actively by buzz pollination, whereas in C and D, it falls out passively. Photos: A, B: Q.C.; C: Plenuska, CC BY-SA 4.0, Wikimedia Commons; D: Amanda Slater, CC BY-SA 2.0, Flickr.

A good example of the disagreements between Müller and Delpino is the *Borago*-type (see Supplementary Data for a translation). This is one of Delpino’s 47 types that brings together a group of plants with the evolutionary convergence of an anther cone, either tight like *Solanum* or a loose cone as in *Galanthus* (Fig. 2). The key feature is that the pollinators (bees) grasp the stamens (graspable structures: ‘apparecchi prensili’), in order to obtain the pollen reward through what are generally poricidal or subporicidal anthers. This insight of Delpino’s is the result of careful empirical observation, and Delpino was a brilliant observer. Of course, there are differences: pollen delivery in *Solanum* is active via buzz pollination; in *Galanthus* it is passive and punctiform; in *Borago* (Faegri, 1986) it is passive and a cloud of pollen is released. Müller sees only differences, whereas Delpino sees them all as ‘graspable structures’ requiring contact between a bee and the androecium.

Most problematic is Müller’s strange contention that *Solanum* is largely, even fundamentally, fly-pollinated. In describing the *Borago*-type, Delpino repeatedly had to refute dubious inferences of fly pollination by Müller. Under *Solanum dulcamara*, he writes: ‘Müller observed no insects visiting the flowers except *Rhingia rostrata* [a fly]. However, this visit is an insignificant accident. We observed the flowers visited by several bumblebees, especially *Bombus italicus*.’ Müller objected, and in his critique, wrote, ‘Delpino … explains those cases in which other insects play substantial roles as agents of cross-pollination, for example the pollen-eating hoverflies at our *Solanum* species, as pure chance events without any meaning. That in this way, through his preconceived opinion, he closes his mind to a deeper understanding of actual facts, can clearly be shown precisely by *Solanum dulcamara*.’ This is an issue that needs to be resolved with quantitative observation, not rhetoric.

In his later paper on the nectary of *Galanthus* (Delpino, 1887), Delpino repeats his commitment to this grouping: ‘The flowers of this Amaryllid [*Galanthus*], classifiable among the grasping mechanisms, belong to the borage type. The flowers of this type are exclusively designated for visitation by bees … The long anthers, attached to robust and short filaments, are connivent with each other, and form a pyramid in whose axis the style passes. They dehisce at the apex through pores, or the dehiscence of the porose type gradually becomes longitudinal. The pollen is dry and smooth, and at the moment when the pollinator clings to the anther pyramid, it necessarily falls onto its sternum. Thus, the pollination area is punctiform, central, sternotribic.’ Notice how Delpino is emphasizing in his classification the functional aspect of the anther cone as a ‘graspable structure’ [*apparecchi prensili*], pointing to the very important observation that the convergent evolution of the anther cone is associated with it being grasped by bees. This insight was completely lost on Müller.

## CONCLUSION: WHAT DO WE DO NOW?

Delpino’s two classifications (as noted above) are intended as groups of species sharing floral traits of functional significance. Later, Delpino’s first functional type classification (Delpino 1) was inverted to become a classification of one trait type (pollination mode) on the basis of other trait types, so becoming ‘pollination syndromes’. The pollination syndrome concept has definite applications and interest, but lacks the advantages of a functional trait classification. However, we cannot blame Delpino for what others did with his classification.

Delpino’s functional type classifications were very early and groundbreaking examples of functional type classification in ecology. Later, vegetation ecologists went in similar directions, an example being the classification of Raunkiaer (Raunkiaer, 1934), in which he differentiated plants according to life form (phanerophytes, hemicryptophytes, etc.). Later still, vegetation ecologists realized the importance of PFTs in global change biology, and functional types, and their uses, generated a huge literature (McMahon *et al*., 2011; Díaz *et al*., 2016; Calbi *et al*., 2024). Functional types can be derived locally, as exemplified by a study (Diaz and Cabido, 1997) identifying PFTs along a climatic gradient in Argentina: 24 traits were measured for the most abundant 100 species and, on the basis of multivariate ordination (DCA) and numerical classification (TWINSPAN), eight functional types were differentiated. Alternatively, PFTs can be defined on a global scale (Harrison *et al*., 2010; Díaz *et al*., 2016).

PFTs are classified using trait combinations. An enormous plant trait database for vegetation science has been instituted by the TRY project, run by the German Centre for Integrative Biodiversity Research (iDiv), and individual trait datasets are increasingly made available, such as by the Open Traits Network (Gallagher *et al*., 2020). A trait-based project related to flowers has been the eFlower project (Sauquet *et al*., 2017). Increasingly, pollination ecology too is moving to be an empirically trait-based science, as envisaged by Delpino and Müller in their respective classifications. Of many examples of recent thinking, a few illustrative studies can be cited here (Goulnik *et al*., 2021; Opedal, 2021; Adedoja and Mallinger, 2024; Peralta *et al*., 2024). Following the lead of PFT classifications, FFTs could be classified in the same way (Table 1). Indeed, pollination ecology might look carefully at the functional trait classification in the *Ulteriori osservazioni* (Delpino, 1873–1874), and even in *Alpenblumen* (Müller, 1881), for inspiration on how to move the field forward.

As Delpino introduced the idea of functional trait classification so early, he gave floral ecology a head start in becoming a fully empirical trait-based science, a head start that was arguably wasted by the side-track to pollination syndromes. One path that floral ecology could take is to catch up and replace pollination syndromes with FFTs.

## Supplementary Material

mcaf197_Supplementary_Data

## Data Availability

No primary data were used in this Botanical Briefing. A translation of Delpino’s ‘*Borago*-type’, as well as table summaries of the three classifications discussed here (Delpino’s first and second classifications, and Müller’s classification), are available in the Supplementary Data.

## References

[mcaf197-B1] Adedoja OA, Mallinger RE. 2024. Can trait matching inform the design of pollinator-friendly urban green spaces? A review and synthesis of the literature. Ecosphere 15: 17. doi:10.1002/ecs2.4734

[mcaf197-B2] Anon . 1883. Hermann Müller [obituary]. Science 2: 487–488. doi:10.1126/science.ns-2.36.487 17756163

[mcaf197-B3] Calbi M, Boenisch G, Boulangeat I, et al 2024. A novel framework to generate plant functional groups for ecological modelling. Ecological Indicators 166: 112370. doi:10.1016/j.ecolind.2024.112370

[mcaf197-B4] Dellinger AS . 2020. Pollination syndromes in the 21^st^ century: where do we stand and where may we go? The New Phytologist 228: 1193–1213. doi:10.1111/nph.16793 33460152

[mcaf197-B5] Delpino F . 1873–1874. Ulteriori osservazioni e considerazioni sulla Dicogamia nel regno vegetale. Atti della Società Italiana di Scienze Naturali 16–17: 151–349, 266–407.

[mcaf197-B6] Delpino F . 1887. Sul nettario florale del *Galanthus nivalis*, L. Malpighia: rassegna mensuale di botanica 1: 354–358.

[mcaf197-B8] Diaz S, Cabido M. 1997. Plant functional types and ecosystem function in relation to global change. Journal of Vegetation Science 8: 463–474. doi:10.2307/3237198

[mcaf197-B7] Díaz S, Kattge J, Cornelissen JHC, et al 2016. The global spectrum of plant form and function. Nature 529: 167–171. doi:10.1038/nature16489 26700811

[mcaf197-B9] Faegri K . 1986. The solanoid flower. Transactions of the Botanical Society of Edinburgh 45: 51–59. doi:10.1080/03746608608684993

[mcaf197-B10] Faegri K, van der Pijl L. 1966. The principles of pollination ecology, 1st edn. Oxford: Pergamon Press.

[mcaf197-B11] Fenster CB, Armbruster WS, Wilson P, Dudash MR, Thomson JD. 2004. Pollination syndromes and floral specialization. Annual Review of Ecology, Evolution, and Systematics 35: 375–403. doi:10.1146/annurev.ecolsys.34.011802.132347

[mcaf197-B12] Gallagher RV, Falster DS, Maitner BS, et al 2020. Open science principles for accelerating trait-based science across the tree of life. Nature Ecology & Evolution 4: 294–303. doi:10.1038/s41559-020-1109-6 32066887

[mcaf197-B13] Goulnik J, Plantureux S, Dajoz I, Michelot-Antalik A. 2021. Using matching traits to study the impacts of land-use intensification on plant-pollinator interactions in European grasslands: a review. Insects 12: 680. doi:10.3390/insects12080680 34442246 PMC8396669

[mcaf197-B14] Harrison SP, Prentice IC, Barboni D, Kohfeld KE, Ni J, Sutra JP. 2010. Ecophysiological and bioclimatic foundations for a global plant functional classification. Journal of Vegetation Science 21: 300–317. doi:10.1111/j.1654-1103.2009.01144.x

[mcaf197-B15] Johnson SD, Wester P. 2017. Stefan Vogel’s analysis of floral syndromes in the South African flora: an appraisal based on 60 years of pollination studies. Flora 232: 200–206. doi:10.1016/j.flora.2017.02.005

[mcaf197-B16] Knuth P . 1898. Handbuch der Blütenbiologie. Leipzig: Wilhelm Engelmann.

[mcaf197-B17] Knuth P . 1906. Handbook of flower pollination [tr. J.R. Ainsworth Davis]. Oxford: Clarendon Press.

[mcaf197-B18] Loew E . 1895. Einführung in die Blütenbiologie auf historischer Grundlage. Berlin: Ferd. Dümmlers Verlagsbuchhandlung.

[mcaf197-B19] McMahon SM, Harrison SP, Armbruster WS, et al 2011. Improving assessment and modelling of climate change impacts on global terrestrial biodiversity. Trends in Ecology & Evolution 26: 249–259. doi:10.1016/j.tree.2011.02.012 21474198

[mcaf197-B20] Müller H . 1873. Die Befruchtung der Blumen durch Insekten. Leipzig: Wilhelm Engelmann.

[mcaf197-B21] Müller H . 1874. C. Befruchtungs- und Aussäungs- Einrichtungen. Botanischer Jahresbericht 2: 880–906.

[mcaf197-B22] Müller H . 1881. Alpenblumen, ihre Befruchtung durch Insekten: und ihre Anpassungen an dieselben. Leipzig: Wilhelm Engelmann.

[mcaf197-B23] Müller H . 1882. Weitere Beobachtungen über Befruchtung der Blumen durch Insekten: III. Verhandlungen des naturhistorischen Vereines der preussischen Rheinlande und Westfalens 39: 1–104.

[mcaf197-B24] Müller H . 1883. The fertilisation of flowers [tr. D’Arcy Wentworth Thompson]. London: Macmillan and Co.

[mcaf197-B25] Ollerton J, Alarcón R, Waser NM, et al 2009. A global test of the pollination syndrome hypothesis. Annals of Botany 103: 1471–1480. doi:10.1093/aob/mcp031 19218577 PMC2701765

[mcaf197-B26] Opedal OH . 2021. A functional view reveals substantial predictability of pollinator-mediated selection. Journal of Pollination Ecology 30: 273–288. doi:10.26786/1920-7603(2021)673

[mcaf197-B27] Peralta G, CaraDonna PJ, Rakosy D, et al 2024. Predicting plant—pollinator interactions: concepts, methods, and challenges. Trends in Ecology & Evolution 39: 494–505. doi:10.1016/j.tree.2023.12.005 38262775

[mcaf197-B28] Raunkiaer C . 1934. The life forms of plants and statistical plant geography. Being collected papers of C. Raunkiaer, translated into English by H. G. Carter, A. G. Tansley, M. S. Fausboll. Oxford: Clarendon Press.

[mcaf197-B29] Sauquet H, von Balthazar M, Magallón S, et al 2017. The ancestral flower of angiosperms and its early diversification. Nature Communications 8: 10. doi:10.1038/ncomms16047 PMC5543309 28763051

[mcaf197-B30] van der Pijl L . 1961. Ecological aspects of flower evolution. II. Zoophilous flower classes. Evolution; International Journal of Organic Evolution 15: 44–59. doi:10.2307/2405842

[mcaf197-B31] Vogel S . 1954. Blütenbiologische Typen als Elemente der Sippengliederung: dargestellt anhand der Flora Südafrikas. Botanische Studien [Fischer, Jena] 1: 1–338.

[mcaf197-B32] Waser NM, Chittka L, Price MV, Williams NM, Ollerton J. 1996. Generalization in pollination systems, and why it matters. Ecology 77: 1043–1060. doi:10.2307/2265575

[mcaf197-B33] Waser N, Ollerton J, Erhardt A. 2011. Typology in pollination biology: lessons from an historical critique. Journal of Pollination Ecology 3: 1–7. doi:10.26786/1920-7603(2011)2

[mcaf197-B34] Werth E . 1915. Kurzer Überblick über die Gesamtfrage der Ornithophilie. Botanische Jahrbücher für Systematik, Pflanzengeschichte und Pflanzengeographie 53: 313–378.

