# Supplementary material for: Beyond pollination syndromes? Reflections on the classifications of Federico Delpino
Source: Ann Bot. 2025 Aug 25;137(1):19–24. doi: 10.1093/aob/mcaf197 (PMC12784065; doi:10.1093/aob/mcaf197)
Supplement: mcaf197_Supplementary_Data [file mcaf197_supplementary_data.docx]

**Supplementary Information**

**Additional Resource and Tables for:**

Beyond pollination syndromes? Reflections on the classifications of Federico Delpino

**Article type:** Botanical briefing

**Author:** Quentin Cronk

**Supplementary Resource**

Translation of “*Borago*-type” from Delpino (1873-1874)

“TWELFTH CLASS.

**GRASPABLE STRUCTURES.**

*Characteristics*. In this class of floral structures, the rather short and converging stamens and styles form a group to which pollinators cling, either to collect pollen or to collect both pollen and nectar simultaneously. In doing so, they inevitably pollinate their sternum, performing cross-pollination with great ease. The colours of the flowers are ordinary; their scents are almost always absent or faintly pronounced. We distinguish two truly natural types within these apparatuses, both bee-pollinated, namely the borage type and the mullein type.

**35. BORAGO-TYPE.**

*Characteristics*. The flowers are pendulous or nearly pendulous, regular. The long anthers, attached to robust and short stamens, converge with one another, forming a pyramid [anther cone] through whose axis the style passes. They dehisce at the apex through pores, or gradually the poricidal dehiscence becomes longitudinal. The pollen is dry and smooth, and the moment a pollinator grasps the anther pyramid, the pollen inevitably falls onto its sternum. Thus, the pollination area is punctiform, central, and sternotribic. For the pollinators’ grasping to occur, the floral expansion is considerable, and generally, the flowers are rosaceous or wheel-shaped. Nectar is sometimes absent, sometimes present but very scarce. In the former case, female bees collect only pollen. This type is exclusively bee-pollinated and, remarkably, recurs with its essential characteristics in many plant families, namely the *Boraginaceae, Primulaceae, Solanaceae, Scrophulariaceae, Amaryllidaceae, Asparagaceae, Pittosporaceae*, and *Loasaceae*.

*Borago officinalis*. Wheel-shaped, pendulous flowers. The anther pyramid, designed to ensure repeated pollinator visits, is reinforced by five rigid filament outgrowths. Anther dehiscence is initially poricidal, then gradually longitudinal. The primary pollinator is the common bee.

*Cyclamen europaeum*, *coum*, *persicum*, and other species. Wheel-shaped, pendulous flowers. Anther dehiscence is initially poricidal, then longitudinal.

*Dodecatheon meadia*. Plukenet (*Almagestum*, p. 62, plate 79, fig. 6), with a beautiful and prescient expression, describes this species: “Virginian bear’s ear with flowers resembling borage, beaked, and reflexed in the manner of cyclamens.”

*Dodecatheon integrifolium*. As the previous species.

*Solanum dulcamara*. A fine example of the type. Pendulous flowers. Completely lacking nectar. Anthers biporose. Dry pollen. Müller (Fertilization of Flowers by Insects, 1873, p. 275) observed no insects visiting the flowers except *Rhingia rostrata*. However, this visit is an insignificant accident. We observed the flowers visited by several bumblebees, especially *Bombus italicus*.

*Solanum nigrum*. Müller (loc. cit.) notes two fly visitors, *Melithreptus* [*Sphaerophoria*] *scriptus* and *Syritta pipiens*. But this is a mere accident. Sprengel (Das entdeckte Geheimnis, p. 129) observed bees and bumblebees as pollinators.

*Solanum tuberosum*. Exclusively bee-pollinated, although Müller (loc. cit.) incidentally observed *Eristalis tenax* and *Syritta pipiens* in the flowers.

*Solanum lycopersicum*, *S. insanum*, and many other species of the genus. All exhibit the borage type; thus, they are exclusively bee-pollinated.

*Verbascum lychnitis*. Apparatus identical to that of *Solanum dulcamara*, but with larger flowers.

*Galanthus nivalis*. Bell-shaped flowers, otherwise exhibiting all other characteristics of the type. Sprengel (loc. cit., pp. 177–180) mentions nectar secreted by the three inner petals. In Vallombrosa specimens, this secretion was entirely absent, according to our repeated and careful observations.

*Leucojum vernum*. Larger bell-shaped flowers; otherwise like the previous species.

*Conanthera bifolia*. Another *Amaryllidaceae* species to be recorded here. It remarkably replicates the floral type of *Cyclamen*.

*Cajophora lateritia* and many *Loasa* species. Borage floral type, but large, with a highly complex nectar-producing apparatus; with a very robust parastaminal pyramid, to which fertile stamens successively approach and recede. Exclusively bee-pollinated and suited to robust, large female bees.

*Sollya linearis*. This Pittosporaceae species also bears flowers characteristic of the borage type.

*Dianella coerulea* and other species of the genus. Recorded here due to their converging, biporose anthers forming a pyramid, and dry, deciduous pollen from pendulous flowers. However, the spongy, orange-colored balloons at the filament tips, which serve wonderfully as grasping points for female bee pollinators, could align them with the following type. It should not be overlooked that the borage type is closely related to the one that follows [*Verbascum*-type].

Thus far, we have cited simple structures; but sometimes the *Borago*-type, or at least a highly analogous type, can be realised in composite flowers, as seen in *Prenanthes purpurea*. Its capitula are five-flowered, pendulous, with highly exserted syngenesious columns. The ensemble of these columns functions equivalently to the anther pyramid of borage-type flowers and serves as a grasping point for bees and bumblebees that avidly visit the flowers, as we observed multiple times at Vallombrosa. The pollen deposition area here, too, is punctiform, central, and sternotribic.”

**Supplementary Table S1.**

A summary of Delpino’s first classification, as published by Müller (Müller, 1873) and the translated version (Müller, 1883).

| A. Plants pollinated through the agency of water. *Hydrophilae* |  |  |
| --- | --- | --- |
| B. Plants pollinated through the agency of wind. *Anemophilae* |  | See Table S2. |
| C. Plants pollinated through the agency of small animals. *Zoidiophilae* | I. *Ornithophilae,* plants adapted for pollination by birds |  |
|  | II. *Entomophilae*, plants adapted for pollination by birds | 1) *Melittophilae*, plants adapted for pollination by (larger) bees |
|  |  | 2) *Micromelittophilae*, plants adapted for pollination by small bees (and a variety of other small insects) |
|  |  | 3) *Myiophilae*, plants adapted for pollination by various Diptera |
|  |  | 4) *Micromyiophilae*, plants adapted for pollination by tiny Diptera |
|  |  | 5) *Sapromyiophilae*, plants adapted for pollination by carrion and dung flies |
|  |  | 6) *Cantharophilae*, plants adapted for pollination by beetles |
|  |  | 7) *Psychophilae*, plants adapted for pollination by daytime butterflies |
|  |  | 8) *Sphingophilae*, plants adapted for pollination by hawkmoths (Sphingidae) |
|  | III. *Malacophilae* plants adapted for pollination by gastropods |  |

**Supplementary Table S2.**

Classification of wind pollinated plants from Delpino’s first classification (Table 1) as summarised by Müller (Müller, 1873).

| **Division** | **Subdivision** | **Type** |
| --- | --- | --- |
| **Wind-pollinated plants (*Anemophilae*)**  Common positive characteristic: only the smooth, easily dispersible texture of the pollen  Common negative characteristics, the absence of brightly colored flower envelopes, fragrance, and nectar | The absence of a stigma distinguishes the **wind-pollinated Gymnosperms**. |  |
|  | **The wind-pollinated Angiosperms**, on the other hand, mostly have greatly developed stigmas that protrude freely as long tails, brushes etc; their male flowers are seldom immobile; mostly they can be shaken by the wind, either because the axes of the male flower clusters, the stalks of the male flowers, or the stamens themselves are long and loosely hanging; in some cases, the flowers elastically release their entire pollen into the air through rapidly snapping anthers. Accordingly, the following five basic forms can be distinguished: | a) Catkin form (*typus amentiflorus*) with mobile axes of the male flower clusters. *Corylus*, *Betula*, etc. |
|  |  | b) Form with hanging flowers (t. *penduliflorus*). *Acer negundo*, *Rumex*. |
|  |  | c) Form with long stamens (t. *longistamineus*). This includes the most numerous wind-flowering plants, e.g., almost all Grasses, Cyperaceae, Juncaceae, *Cannabis*, *Humulus*, *Empetrum*, *Mercurialis, Ricinus, Plantago, Littorella, Callitriche, Myriophyllum, Hippuris*, the wind-flowering *Thalictrum* species, and others. |
|  |  | d) Rapidly releasing form (t. *explodens*). *Urtica, Parietaria, Morus*. |
|  |  | e) Form with immobile flowers (t. *immotiflorus*). This includes many palms, *Potamogeton, Triglochin, Sparganium, Typha*. |

**Supplementary Table S3.**

Delpino’s second classification as summarised by (Knuth, 1906). This classification was heavily criticised by Müller, but defended by Löw (Loew, 1895) (see text).

| **CLASS** | **SUBCLASS** |
| --- | --- |
| Class I. Arrangements for temporary imprisonment. (*Apparecchi a carcere temporaria*). The visitors fall into a cavity, and remain for a time in captivity. | 1. Aristolochia type (Micromyophilous form, Sapromyophilous form). 2. Cypripedium type. 3. Coryanthes type |
| Class II. Lodger arrangements. (*Apparecchi a ricovero*). The visitors voluntarily spend some time in the flowers that protect them. | 4. Aspidistra type (Micromyophilous form, Sapromyophilous form). 5. Magnolia type (Beetle flowers). 6. Hydrangea type (Beetle flowers.). 7. Fig type. |
| Class III. Tubular arrangements. (*Apparecchi tubati*). The visitors enter the wide corolla tube, only remaining there long enough to gather pollen or suck nectar. | 8. Datura type. 9. Campanula type. 10. Digitalis type. (sternotribous form, nototribous forin. |
| Class IV. Pendulous arrangements. (*Apparecchi pendoloni*). The visitors partly or entirely enter the pendulous flowers. | 11. Fuchsia type, 12. Abutilon type. |
| Class V. Small-mouthed flowers. (*Apparecchi microstomi*). Owing to the narrowness of the corolla, visitors can only introduce their sucking organs: frequently ornithophilous. | 13. Microstomous type. |
| Class VI. Labiate arrangements. (*Apparecchi labiati*). Bilaterally symmetrical flowers with nectaries on the under side, while anthers and stigmas are on the upper side. Visitors (bees, birds) touch the anthers and stigmas with their backs. | 14. Labiate type (galeate form, ringent form, personate form, labiate form, unilabiate form). 15. Aeschynanthus type. 16. Violet type. |
| Class VII. Papilionaceous arrangements. (*Apparecchi papilionacei*). The visitors touch the anthers and stigma of the bilaterally symmetrical flowers with their ventral surface. | 17. Normal papilionaceous type, with concealed anthers (common papilionaceous form, tension form, pump form, brush form). 18. Papilionaceous type with exposed stamens. 19. Amaryllis or Rhododendron type (form with stamens completely or almost completely enclosed, form with exserted stamens). 20. Melastomaceous type. 21. Strelitzia type. |
| Class VIII. Narrow-tubed arrangements. (*Apparecchi sifonifori e macrosifoni*). The corolla tubes are long, and often so narrow that only sphingidae can get at the honey. | 22. Long-spurred type. 23. Long-flowered type. |
| Class IX. Arrangements for hovering visitors. (*Apparecchi circumvolatorii*). Sphingidae and birds effect pollination while hovering before the flower. | 24. Methonica type. 25. Stenocarpus type. 26. Crocus type. 27. Protea type. 28. Callistemon type. |
| Class X. Arrangements for wandering visitors. (*Apparecchi perambulatorii*). The visitors (bees) wander about, either on the whole surface to be pollinated, or only on a ring-like zone of it. | 29. Passiflora type. 30. Nigella type. 31. Helianthus type. |
| Class XI. Arrangements for creeping visitors. (*Apparecchi reptatorii*). The visitors (snails) crawl about on the flat inflorescence. | 32. Rhodea type. 33. Anthurium type. 34. Chrysosplenium type. |
| Class XII. Prehensile arrangements. (*Apparecchi prensili*). The visitors grasp the style and stamens in such a way as to cover their breasts with pollen, and then effect cross-pollination. | 35. Borago type. 36. Verbascum type. |
| Class XIII. Regular open arrangements. (*Apparecchi aperti, regolari*). The widely open flowers are visited by insects of the most varied kind (beetles, bees, flies, occasionally butterflies, &c.). | Green open arrangements (*Apparecchi aperti,* cloranti): 37. Rhamnus type. Black open arrangements (*Apparecchi aperti, melananti*): 38. Uvaria type. 39. Stapelia type. 40. Dark type. Many-flowered open arrangements (*Apparecchi aperti, polianti*): 41. Stellate type. 42. Scabious type. 43. Trachelium type. Brightly coloured open arrangements (*Apparecchi aperti, callipetali):* 44. Papaver type. 45. Rosa type. 46. Ranunculus type. Small-flowered open arrangements (*Apparecchi aperti, brachipetali*)*.* 47. Small-flowered type. |

**Supplementary Table S4.** Müller’s floral functional type classification (insect-pollinated flowers of Central Europe), from *Alpenblumen* (Müller, 1881), as simplified from Knuth’s Handbook (Knuth, 1906).

| *Insect-pollinated flowers, Entomophilae, En (Delpino group).* | | |
| --- | --- | --- |
| **Reward** | **Class** | **Subclass** |
| Pollen | 1. Pollenflowers, Po. |  |
| Nectar | 2. Flowers with exposed nectar, E. |  |
|  | 3. Flowers with partly concealed nectar, EC. |  |
|  | 4. Flowers with concealed nectar, C. |  |
|  | 5. Social flowers, S. |  |
|  | 6. Hymenopterid flowers, H. | (a) Bee flowers, Hb. |
|  |  | (b) Humble-bee flowers, Hh. |
|  |  | (c) Bee-humble-bee flowers, Hbh. |
|  |  | (d) Wasp flowers, Hw. |
|  |  | (e) Ichneumon flowers, Hi. |
|  | 7. Lepidopterid flowers, L. | (a) Butterfly flowers. Lb. |
|  |  | (b) Moth flowers, Lm. |
|  | 8. Fly flowers, F. | (a) Nauseous flowers, Fn. |
|  |  | (b) Pitfall flowers, Fpf. |
|  |  | (c) Pinch-trap flowers, Fpt. |
|  |  | (d) Deceptive flowers, Fd. |
|  |  | (e) Hover-fly flowers, Fh. |
|  | 9. Small-insect flowers, Sm.  “Visited and cross-pollinated by small insects belonging to various orders. [E.g.] *Herminium Monorchis* … visited by equally small Hymenoptera, Diptera, and Coleoptera, … tiny Ichneumons (Braconidae and Pteromalidae).” |  |
